# Long-term outcomes after elective inguinal hernia mesh-repair in patients with inflammatory bowel disease

**DOI:** 10.1007/s10029-025-03362-3

**Published:** 2025-05-23

**Authors:** Hans Lovén, Rune Erichsen, Anders Tøttrup, Thue Bisgaard

**Affiliations:** 1 Centre for Surgical Science, Zealand University Hospital, University of Copenhagen, Lykkebækvej 1, Køge, 4600 Denmark; 2 Department of Clinical Epidemiology, Aarhus University Hospital, Aarhus, Denmark; 3 Department of Surgery, Randers Regional Hospital, Randers, Denmark; 4 Department of Surgery, Region Hospital Viborg, Viborg, Denmark; 5 Department of Surgery, North Denmark Regional Hospital, Hjørring, Denmark

**Keywords:** Inguinal hernia repair, IBD, Fistulising disease, Crohn’s disease, Ulcerative colitis, TAPP, Lichtenstein, Recurrence, Meshrelated complications

## Abstract

**Background:**

Knowledge of long-term outcomes following elective inguinal hernia mesh-repair in patients with inflammatory bowel disease (IBD) remains limited. Pathophysiological differences between Crohn’s disease (CD) and ulcerative colitis (UC) may influence mesh-related complications and recurrence risk. The primary objective was to assess the reoperation risk for mesh-related complications, and secondarily, recurrence after inguinal hernia mesh-repair in patients with CD and UC. The impact of fistulising disease (intra-abdominal/perianal) and surgical technique (open/laparoscopic) on both outcomes was also analysed based on the available data.

**Methods:**

This nationwide cohort study (2007–2016) followed IBD patients undergoing elective inguinal hernia mesh-repair to assess risks of reoperation for mesh-related complications or recurrence. Risks were estimated using cumulative incidence and Cox regression analyses.

**Results:**

Among 1,072 patients with IBD (CD = 264, UC = 698, IBD-unclassified = 110), the five-year reoperation risk was 0.5% for mesh-related complications and 5.7% for recurrence. Fistulising disease was present in 6.9% (*n* = 74) of all patients with IBD: perianal in 95% (*n* = 70) and intra-abdominal in 5% (*n* = 4). There were too few mesh-related complications (*n* = 5) to support statistical analysis of this outcome. Recurrence risk was not significantly affected by IBD subtype: CD (reference), UC (HR = 1.67, 95% CI: 0.77–3.64), IBD-U (HR = 0.91, 95% CI: 0.24–3.44), or surgical technique: transabdominal preperitoneal (TAPP) (reference), and Lichtenstein (HR = 0.80, 95% CI: 0.43–1.47).

**Conclusion:**

This study suggests that inguinal hernia mesh-repair is also safe among IBD patients regardless of subtype, surgical technique, or perianal fistulation. Similarly, recurrence risk was unaffected by these factors. Limited data prevented conclusions on intra-abdominal fistulising disease as a potential risk-factor for poor surgical outcomes.

**Supplementary Information:**

The online version contains supplementary material available at 10.1007/s10029-025-03362-3.

## Introduction

Several studies have identified IBD as a risk factor for postoperative complications following abdominal surgery [1–6]. Inguinal hernia mesh-repair is considered a minor surgical procedure and one of the most common abdominal operations. However, there are only a few studies of its long-term outcomes in patients with IBD. Complications related to mesh use, such as enteric fistulas interacting with synthetic mesh implants, can result in chronic mesh infections, repeated reoperations, and potentially fatal outcomes.

This study hypothesised that patients with CD have a higher risk of mesh-related complications and recurrence after elective inguinal hernia mesh-repair compared with UC patients. Pathophysiological differences, including enterocutaneous fistulas may contribute to increased surgical complications such as mesh-related complications in patients with CD.

The primary objective was to compare the risks of reoperation for mesh-related complications in patients with CD, UC, and unclassified IBD (IBD-U). The secondary objective was to estimate the risk of reoperation for recurrence in the same groups. Additionally, the impact of surgical technique and fistulising disease (perianal and intra-abdominal) on mesh-related complications and recurrence was assessed.

## Methods

In this nationwide cohort study (2007–2016), data from three nationwide registers were combined using the unique civil registry number (CPR number) assigned to all Danes and used in all registers. Adherence to the STROBE (Strengthening the Reporting of Observational Studies in Epidemiology) guidelines for observational studies was ensured [7]. 

### Registers and data sources

The Danish Hernia Database (DHDB) has been collecting detailed intraoperative information on inguinal hernia repair since 1998. The DHDB is a highly valid registry covering approximately 90% of hernia repairs in Denmark [8, 9]. Standardised reporting, audits, and validation against clinical records ensures accurate data on hernia type, surgical technique, and complications, making it a reliable tool for evaluating outcomes in a single clinical setting [8–10]. The Danish Civil Registration System (CRS) holds detailed individual-level data on e.g., sex, date of birth, emigration, immigration, disappearance, and death for all Danish citizens. It is widely regarded as highly valid and complete [11]. The National Patient Registry (NPR) has recorded all hospital visits in Denmark since 1977. The validity of IBD diagnosis codes and surgical procedure documentation has been confirmed, ensuring reliable and comprehensive data [12–14]. 

Perioperative details, including mesh-related complications, were manually extracted from the medical records. To address any potential gaps in DHDB registration, supplementary data from the NPR and medical records were included.

### Cohort description

The study included patients diagnosed with IBD from 1996 onwards who subsequently underwent inguinal hernia repair between 1 January 2007, and 31 December 2016. The index operation was defined as the first record of inguinal hernia mesh-repair in DHDB whether it was a primary or recurrent operation. Inguinal hernia repair procedures were categorised as open, laparoscopic, or other. Open mesh repair was defined as Lichtenstein repair, whereas laparoscopic mesh repair was specifically categorised as TAPP (transabdominal preperitoneal inguinal hernia repair). The number of ‘other’ repairs (e.g., open non-mesh repair, other open-mesh repair, plug, laparoscopic TEP [totally extraperitoneal]) was displayed in Table 1 but not included for the subgroup analysis. Although TEP is a recognized laparoscopic technique, it is very rarely performed in Denmark.

**Table 1:** Baseline characteristics of patients with inflammatory bowel disease undergoing elective inguinal hernia repair 2007–2016

	CD	UC	IBD-U	Overall	*P*-value
	(*N* = 264)	(*N* = 698)	(*N* = 110)	(*N* = 1072)	
*Period*					
2007–2009	63 (23.9)	26 (23.6)	196 (28.1)	285 (26.6)	N.S.
2010–2013	112 (42.4)	40 (36.3)	283 (40.5)	435 (40.6)	
2014–2016	89 (33.7)	44 (40.0)	219 (31.4)	352 (32.8)	
*Age*					
Median (range)	62 (19–93)	63 (18–90)	54 (30–88)	62 (18–93)	N.S.
*Sex*					
Female	44 (16.7)	96 (13.8)	19 (17.3)	159 (14.8)	N.S.
Male	220 (83.3)	602 (86.2)	91 (82.7)	913 (85.2)	
*Charlson Comorbidity Index*					
0–2	239 (90.5)	663 (95.0)	104 (94.5)	1006 (93.8)	N.S.
> 3	25 (9.5)	35 (5.0)	6 (5.5)	66 (6.2)	
*Index operation*					
Primary	245 (92.8)	632 (90.5)	104 (94.5)	981 (91.5)	N.S.
Recurrent	19 (7.2)	66 (9.5)	6 (5.5)	91 (8.5)	
Type of hernia					
Inguinal	248 (93.9)	657 (94.1)	104 (94.5)	1009 (94.1)	N.S.
Femoral	12 (4.5)	38 (5.4)	4 (3.6)	54 (5.0)	
Combination ^c^	4 (1.5)	3 (0.1)	2 (1.8)	7 (0.7)	
*Type of repair*					
TAPP	74 (29.4)	209 (31.7)	32 (30.2)	315 (30.9)	N.S.
Lichtenstein	164 (65.1)	438 (66.4)	70 (66.0)	672 (66.0)	
Other ^a^	26 (9.8)	51 (7.3)	8 (7.3)	85 (7.9)	
*Use of Anti-TNF-alfa*					
Yes	30 (11.4)	22 (3.2)	17 (15.5)	69 (6.4)	< 0.005
No	234 (88.6)	676 (96.8)	93 (84.5)	1003 (93.6)	
*Fistulising disease* ^b^					
Yes	31 (11.7)	27 (3.9)	16 (14.5)	74 (6.9)	< 0.005
No	233 (88.3)	671 (96.1)	94 (85.5)	998 (93.1)	
*Type of fistula*					
Isolated perianal	27 (87.1)	27 (100)	16 (100)	70 (94.5)	N.S.
Intra-abdominal	4 (12.9)	0 (0)	0 (0)	4 (5.5)	
*IBD duration*					
Median (range) years	8.5 (0–34.0)	9.0 (0–24.0)	8 (0–39.0)	8.0 (0–39.0)	N.S.

^a^ Includes other open-mesh repair (*n* = 15), plug (*n* = 6), open non-mesh repair (*n* = 8), other laparoscopic repair (*n* = 20), and non-specified (*n* = 36)

^b^ Includes any type of fistula (i.e., perianal and non-perianal fistulas)

^c^ Both femoral and inguinal hernia

### Definition of IBD subtypes and fistulising disease

The methodology used to define CD, UC, IBD-U, and fistulising disease was consistent with that used in our previous study [15] and is detailed in Supplementary Appendix 1. Fistulising disease was defined using surgical and diagnostic codes from the NPR, occurring before start of the study, and further subdivided into isolated perianal or intra-abdominal manifestations (Supplementary Appendix 1).

Diagnosis and surgical procedure codes were classified based on the International Classification of Diseases, Tenth Revision (ICD-10) and the Nordic Medico-Statistical Committee classification for surgical procedures.

### Outcomes

Mesh-related complications and recurrence were defined as relevant reoperations occurring from 30 days after the index operation until the end of follow-up. Two investigators (HL and TB) manually reviewed the patients’ medical records for all abdominal reoperations during follow-up to determine their association with the inguinal hernia mesh-repair. Reoperations were further categorised as ‘not mesh-related’, ‘possibly mesh-related’, and ‘definitely mesh-related’. Only cases with ‘possible’ and ‘definite’ relationships were included. The investigators were blinded to IBD subtypes and hernia mesh-repair techniques. The severity of mesh-related complications was classified using the Clavien-Dindo system for surgical complications [16]. Reoperation for recurrence was only valid when performed on the same side as the index operation.

### Covariates

Data on sex, age, comorbidities, presence of fistulising disease, and use of anti-TNF-α medications were obtained from the NPR. The use of anti-TNF-α medication was documented if it had been administered within three months before the date of inguinal hernia mesh-repair. The Charlson Comorbidity Index (CCI) [17] assessed patients’ comorbidity status, with relevant ICD codes from the NPR indicating specified diseases within the CCI scoring system. All covariates and outcome variables were defined prior to the start of the study. Perioperative hernia-related details were obtained from DHDB and included the type of inguinal hernia (inguinal/femoral/combination), index operation (primary/recurrent), and type of hernia repair (open/laparoscopic/other). The time periods were divided into three intervals to accommodate potential shifts in surgical technique, mesh materials, and guideline updates during the study period.

### Follow-up

Follow-up was ensured through the comprehensive nature of NPR, as outlined above, supplemented by a manual review of all abdominal reoperations during the follow-up period. Denmark’s very low emigration rate and the exclusion of patients with missing medical records further guaranteed 100% follow-up for all the included patients.

### Exclusion criteria

**Fig. 1:**
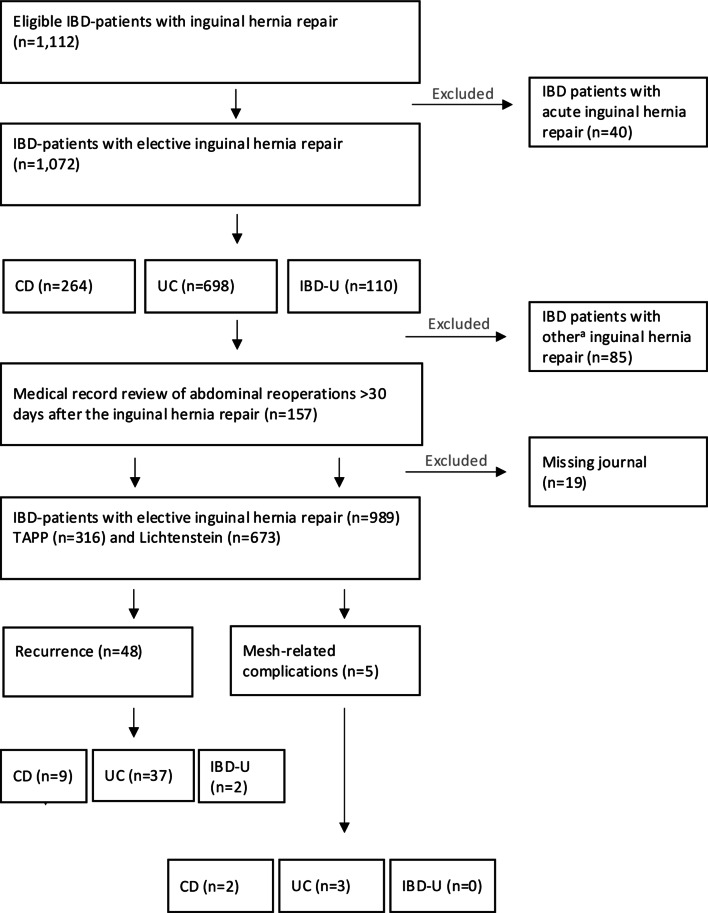
Inclusion of patients IBD and reported outcomes after inguinal hernia repair. ^a^ Includes other open-mesh repair (n=15), plug (n=6), open non-mesh repair (n=8), other laparoscopic repair (n=20), and non-specified (n=36)

Patients undergoing acute inguinal hernia repair, those operated on with techniques other than Lichtenstein and TAPP, and those younger than 18 years were excluded from the study. Additionally, patients with missing medical records for complication review but with evidence of reoperation in the NPR were also excluded (Fig. 1).

### Statistics

Continuous variables were reported as medians and ranges, whereas categorical variables were reported as frequencies and percentages. The median follow-up duration was calculated using the reverse Kaplan-Meier method and presented as median and interquartile range (IQR). Follow-up was initiated 30 days after the first registered elective inguinal hernia mesh-repair in DHDB and continued until the date of reoperation for mesh-related complications or recurrence, death, emigration, or the end of follow-up. The risk of reoperation for mesh-related complications and recurrence was calculated using the cumulative incidence proportion, treating death as a competing risk. Risk was compared between subgroups of patients using either Cox proportional hazard regression, Fisher’s exact test, or Log-Rank test. Cox-Aalen method ensured proportional hazard of the relevant variables. P-values < 0.05 were considered statistically significant.

## Results

A total of 1,072 adult patients with IBD (CD = 264, UC = 698, IBD-U = 110) undergoing inguinal hernia repair were included in the study. The median follow-up period for all IBD patients was 4.3 years (IQR: 2.1–6.9), with no significant differences between CD, UC, and IBD-U (*p* = 0.80). The follow-up rate was 100%, and the median time from inguinal hernia mesh-repair to reoperation for either recurrence or complications was 2.1 years (IQR: 0.9–2.7) for the entire cohort, with no significant differences between the groups (*p* = 0.72).

There were no substantial differences in baseline characteristics (Table 1) between the IBD subtypes, except for the presence of fistulising disease and treatment with anti-TNF-α medication, which were more common in CD and IBD-U than in UC (Table 1). Regardless of the IBD subtype, the majority of patients (90–95%) underwent primary inguinal hernia mesh-repair for an initial hernia, with approximately 65% undergoing open Lichtenstein repair, compared with 35% TAPP. All the patients who underwent mesh repair were treated with various types of synthetic non-absorbable meshes. Thus, there were no patients with biological- or synthetic absorbable mesh implantation. Fistulising disease (both perianal and intra-abdominal) affected 31 (11.7%), 27 (3.9%), and 16 (14.5%) patients with CD, UC, and IBD-U, respectively. Among UC and IBD-U all fistulas were isolated perianal. In CD, four (12.9%) patients had intra-abdominal fistulas and the remaining 27 (87.1%) had isolated perianal fistulas (Table 1).

Five patients (CD, *n* = 2; UC, *n* = 3; IBD-U, *n* = 0) underwent reoperation for mesh-related complications (deep wound infection, *n* = 4; pain, *n* = 1) (Fig. 1). The overall five-year risk of reoperation for mesh-related complications was 0.5% (95% CI: 0–0.9%). All mesh-related complications were classified as Clavien-Dindo grade III, and the mesh was removed in all cases. These complications occurred in both TAPP (*n* = 2) and Lichtenstein (*n* = 3) procedures. All patients with mesh-related complications (*n* = 5) had non-fistulising disease without anti-TNF-α treatment (*n* = 0).

**Fig. 2:**
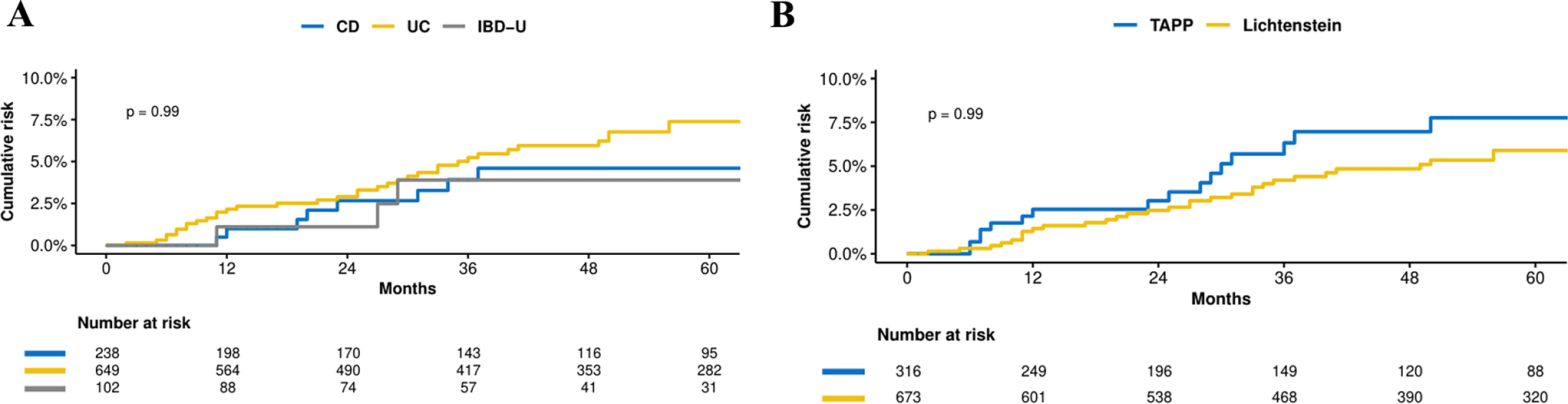
Cumulative risk of reoperation for recurrence after inguinal hernia repair in patients with inflammatory bowel disease. (**A**) Risk of reoperation for recurrence between IBD-subtypes (n=989). (**B**) Risk of reoperation for recurrence between TAPP and Lichtenstein in all IBD patients (n=989)

A total of 48 IBD patients (CD = 9; UC = 37; IBD-U = 2) underwent reoperation for recurrence resulting in an overall five-year risk of 5.7%. Compared with patients with CD, the hazard ratio (HR) of recurrence was 1.67 (95% CI: 0.78–3.58) among UC, and 0.91 (95% CI: 0.24–3.44) among IBD-U patients. The HR of recurrence was 0.80 (95% CI: 0.43–1.47) comparing TAPP repair to Lichtenstein technique. Perianal fistulising disease (HR: 1.41, 95% CI: 0.50–4.02) did not influence the risk of recurrence (Fig. 2A and B; Table 2).

**Table 2:** Risk of reoperation for recurrence in patients with IBD using Cox regression analysis

		HR (univariable)	HR (multivariable) ^a^
*IBD subtype*			
CD	238 (24.1)	1.0 (reference)	1.0 (reference)
UC	649 (65.6)	1.67 (95% CI: 0.78–3.58, *p* = 0.190)	1.67 (95% CI: 0.77–3.64, *p* = 0.198)
IBD-U	102 (10.3)	0.94 (95% CI: 0.25–3.53, *p* = 0.923)	0.91 (95% CI: 0.24–3.44, *p* = 0.891)
Total	**989 (100)**		
*Approach*			
TAPP	316 (31.9)	1.0 (reference)	1.0 (reference)
Lichtenstein	673 (68.1)	0.80 (95% CI: 0.43–1.47, *p* = 0.472)	0.80 (95% CI: 0.43–1.47, *p* = 0.470)
*Fistulising disease*			
No	920 (93.2)	1.0 (reference)	1.0 (reference)
Yes	69 (6.8)	1.16 (95% CI: 0.42–3.24, *p* = 0.772)	1.41 (95% CI: 0.50–4.02, *p* = 0.520)

^a^ Adjusted for IBD subtype, surgical approach and fistulising disease

## Discussion

In this nationwide cohort study with 100% long-term follow-up, the absolute risk of mesh-related complications was low, regardless of IBD subtype, surgical technique, or presence of perianal fistulas. Similarly, recurrence risk was unaffected by these factors. Unfortunately, the data did not permit conclusions regarding intra-abdominal fistulising disease as a potential risk factor for poor surgical outcomes.

In the present study, the risk of mesh-related complications in patients with IBD was not different to that reported in the non-IBD specific population [18]. Hence, a 2007 retrospective study (not specific to IBD, *n* = 1,452) [18] reported a five-year risk of 0.35% for late-onset deep mesh infection following inguinal hernia mesh-repair. Due to the limited number of mesh-related complications in the present study, comparing risks between CD, UC, and IBD-U or between TAPP and Lichtenstein repairs was not feasible. However, the low absolute risk of long-term mesh-related complications, irrespective of IBD subtype or surgical technique, indicates that inguinal hernia repair using a synthetic non-absorbable mesh is safe also among patients with IBD.

The five-year risk of hernia recurrence in non-IBD populations is typically 2–4% [19, 20], but this study found a higher risk of 5.7% regardless of IBD subtype or surgical technique. It is speculated that this higher risk is likely due to IBD-specific factors such as chronic inflammation, impaired collagen metabolism, immunosuppressive therapy, and non-surgical postoperative complications. These factors weaken tissue repair and increase the recurrence risk [21], consistent with previous findings of higher short-term complications in IBD patients after hernia repair [22]. 

The incidence of fistulising CD varies between 12% and 50% depending on the type of fistula and CD duration [23]. In this study, fistulising disease was present in 11% of CD patients and 3.9% of UC patients, consistent with the literature [24]. The occurrence in IBD-U was 14.5%, likely reflecting longer disease duration with greater risk of mixed diagnosis codes. A previous study from 2024 [15] found that CD patients with intra-abdominal fistulising disease had a significantly higher risk of mesh-related complications after incisional hernia mesh-repair compared to those with isolated perianal disease. However, few patients with a history of intra-abdominal fistulising disease were included in the current study, likely because those with more severe IBD and higher comorbidities required more extensive IBD-related surgeries, making inguinal hernia repair less of a priority.

The present study had both strengths and limitations. Its register-based design and the high completeness of the registers, including complete follow-up, contributed to a low risk of selection bias and ensured generalisability and a high degree of extern validity. Also, misclassification of exposure and outcome variables was minimal [14, 25]. The main limitation of this study was related to the limited number of outcome events, leading to reduced statistical power. Consequently, it was not possible to conduct subgroup analyses or measure the impact of covariates on all defined outcomes. Unfortunately, the dataset used for this study did not include a well-defined non-IBD comparison cohort. While a comparison with non-IBD patients would indeed be informative, this was beyond the scope of our current analysis.

Although the IBD subtypes were similar in most aspects of baseline characteristics, we cannot rule out the impact of unmeasured confounding factors. The data did not include details on smoking habits, BMI, or diabetes, all of which are known risk factors for postoperative complications. However, the comorbidity score measured by the CCI score may to some degree account for these factors. Additionally, detailed data on specific synthetic mesh types used were unavailable. Due to the limited number of cases, a meaningful statistical comparison between different synthetic meshes would not have been feasible.

Future research should aim to explore the risk of mesh-related complications and recurrence following both inguinal- and ventral hernia mesh repair, with a specific focus on patients with intra-abdominal fistulising disease, as well as other potential risk factors. To achieve this, large-scale, multinational, register-based cohort studies are essential to provide robust data and comprehensive insights into these risks and contributing factors.

In conclusion, this study showed that inguinal hernia mesh repair using a non-absorbable synthetic mesh also seems safe among IBD patients — regardless of IBD subtype, surgical technique, or perianal fistulation. Similarly, recurrence risk was unaffected by these factors. However, limited data prevented conclusions on intra-abdominal fistulising disease as a risk factor for poor surgical outcomes.

## Electronic supplementary material

Below is the link to the electronic supplementary material.

Supplementary Appendix 1

## Data Availability

The Danish Health Data Authority provided the data for this article with license/permission, and the corresponding authormay request to receive the data on reasonable terms with the approval of the Danish Health Data Authority.
