# Supplementary material for: Long-term outcomes after elective inguinal hernia mesh-repair in patients with inflammatory bowel disease
Source: Hernia. 2025 May 23;29(1):183. doi: 10.1007/s10029-025-03362-3 (PMC12102129; doi:10.1007/s10029-025-03362-3)
Supplement: Supplementary file 1 — Supplementary Appendix 1 [file 10029_2025_3362_MOESM1_ESM.docx]

**SUPPLEMENTARY APPENDIX 1**

**Definition of Crohn’s disease (CD)**

- Diagnosed exclusively with K50*.

- Patients diagnosed with both UC and CD, but exclusively CD (K50*) diagnosis in the five most recent years.

- Patients diagnosed with both UC and CD or only with UC, but with only typical CD operations as listed below (i.e., no history of colectomy).

| Small bowel resection | KJFB00 |
| --- | --- |
| Laparoscopic small bowel resection | KJFB01 |
| Reverting a segment of small bowel | KJFB10 |
| Extension plastic of small bowel | KJFB13 |
| Ileocecal resection | KJFB20 |
| Laparoscopic ileocecal resection | KJFB21 |

**Definition of ulcerative colitis (UC)**

- Diagnosed exclusively with K51*.

- Patients diagnosed with both UC and CD, but exclusively UC (K51*) diagnosis in the five most recent years.

**Definition of IBD-unclassified (IBD-U)**

- Diagnosed with both CD (K50*) and UC (K51*) codes.

**Definition of intra-abdominal fistulising disease**

| Cutaneous fistula | DL984 |
| --- | --- |
| Bowel abscess | DK630 |
| Bowel fistula | DK632 |
| Closure of a fistula to the small bowel | KJFA76 |
| Closure of a colonic fistula | KJFA86 |

**Definition of perianal fistulising disease**

| Anal fistula | DK603 |
| --- | --- |
| Rectal fistula | DK604 |
| Anorectal fistula | DK605 |
| Anal abscess | DK610 |
| Rectal abscess | DK611 |
| Anorectal abscess | DK612 |
| Ischiorectal abscess | DK613 |
| Intrasphincteric anal abscess | DK614 |
| Anal or rectal stenosis | DK624 |
| Anovaginal fistula | N822, N823A, N824 |
| Anal fissure | DK600, DK601, DK602 |
| Lay open or incision of perianal fistula | KJHD20 |
| Excision of perianal fistula | KJHD23 |
| Partial lay open or excision of perianal fistula (including seton placement) | KJHD30 |
| Completion lay open or excision of perianal fistula | KJHD33 |
| Endoscopic guided thermic destruction of anal fistula | KJHD42 |
| Closure of anal fistula with laser | KJHD43 |
| Injection of stem cells in anal fistula | KJHD46 |
| Occlusion of perianal fistula with collagen plug | KJHD60 |
| Closure of anal fistula with clips | KJHD63 |
| Ligature of anal fistula | KJHD66 |
| Other operation of anal fistula | KJHD99 |
| Partial lay open or excision of perianal fistula (including seton placement) | KJHD30 |
